# Prevalence of tobacco smoking and its association with disease severity and psoriatic arthritis among patients with nail psoriasis in China: a cross-sectional study

**DOI:** 10.3389/fmed.2025.1660724

**Published:** 2025-12-09

**Authors:** Xuen Yang, Lingyi Lu, Huiying Zhou, Xinyi Xu, Bingjiang Lin

**Affiliations:** 1 Department of Dermatology, The First Affiliated Hospital of Ningbo University, Ningbo, Zhejiang, China; 2 School of Medicine, Ningbo University, Ningbo, Zhejiang, China; 3 The Third People’s Hospital of Beilun District, Ningbo, China; 4 National Clinical Research Center for Dermatologic and Immunologic Diseases, Beijing, China

**Keywords:** psoriasis, nail psoriasis, psoriatic arthritis, smoking status, smoking intensity, smoking index

## Abstract

**Introduction:**

Nail psoriasis is a common, treatment-refractory manifestation of psoriasis. Smoking is a key environmental factor implicated in nail and articular psoriasis. While smoking’s association with cutaneous psoriasis is well-studied, its relationship with nail psoriasis remains less explored.

**Objective:**

The primary objective of this study was to investigate the impact of smoking on the severity of nail psoriasis and arthritic psoriasis in patients with nail psoriasis.

**Methods:**

Data from 1044 nail psoriasis patients within a population-based registry in China were analyzed. We assessed associations of smoking status and intensity with sociodemographics, disease severity and dermatology quality of life measures (including PASI, BSA, DLQI, and PEST scores), and PsA diagnosis. Analyses used SPSS 29.0; *p* < 0.05 defined significance.

**Results:**

The current smoking rate among patients with nail psoriasis is 34.6%, which is much higher than the current smoking rate among patients without nail psoriasis (20.2%). The proportions of the three different smoking intensities are also much higher than those among patients without nail psoriasis. Current smoking affects the total nail involvement count in patients with nail psoriasis, and severe smoking affects both the total nail involvement count and the nails with > 90% area involvement count in these patients. However, we found that smoking intensity was negatively correlated with DLQI scores in nail psoriasis, which is contrary to previous studies on plaque psoriasis. Spearman’s correlation analysis revealed that smoking intensity and smoking index were positively associated with total nail involvement count and individual nails > 90% with area involvement count. In the regression analysis for PSA, the OR for current smokers was 0.57 (95% CI: 0.35–0.92) compared to non-smokers, and the OR for severe smokers was 0.37 (95% CI: 0.15–0.90) compared to mild smokers.

**Conclusion:**

Patients with nail psoriasis have higher smoking rates and smoking intensity compared to those without nail psoriasis. The total nail involvement count was higher in current smokers than in non-smokers. Smoking intensity was positively associated with total nail involvement count and individual nails with > 90% area involvement count. Current smoking was a negative associated factor for PEST risk level. Both current smoking and severe smoking were negative associations with the presence of psoriatic arthritis.

## Introduction

Psoriasis is a prevalent chronic inflammatory disease, characterized by the appearance of well-demarcated red plaques covered with silvery-white scales. The prevalence of psoriasis in adults ranges from 0.51 to 11.43%, and in China, the prevalence is approximately 0.47% (1). Psoriasis is a multifaceted condition that manifests not only in the form of cutaneous lesions, but also in the form of fingernail (toe) and systemic manifestations. Nail psoriasis has been observed to manifest with a variety of clinical features, including oil-drop discoloration, pitting hemorrhages, subungual hyperkeratosis, and distal subungual separation. The prevalence of nail psoriasis among patients with psoriasis ranges from 50 to 79% (2), and the presence of nail psoriasis without accompanying skin lesions is approximately 5% (2, 3). Nail psoriasis has a significant impact on the quality of life of patients, including psychological and functional impairment. In addition, nail psoriasis is an independent factor in the development of psoriatic arthritis (PsA), with approximately 80% of PsA patients experiencing nail involvement (2). Nail psoriasis has been linked to a variety of factors, including autoimmune, genetic, and environmental influences. In recent years, a growing body of research has identified smoking as a significant environmental factor contributing to the development of nail psoriasis. Yildiz Hayran et al. found that smoking not only exacerbates cutaneous PASI score in psoriasis, but also increases the frequency of nail psoriasis involvement (4). Smoking increases the prevalence of PsA, but some studies have suggested that the development of PsA in psoriasis patients may be negatively associated with smoking (5, 6). Nevertheless, it is currently believed that promoting smoking cessation is crucial to curbing the development of psoriasis and related diseases.

Tobacco products have been shown to be hazardous to health, and smoking is now one of the leading causes of death and disability worldwide. By 2020, the global smoking rates for men and women were 32.6 and 6.5%, respectively. Over the past 40 years, smoking rates have declined annually, with decreases of over 40% in developed countries. However, smoking rates have remained largely unchanged in most developing countries, and over half of men in China continue to smoke (7). The smoking index (SI) is an important indicator of an individual’s smoking status and intensity. The SI quantifies the cumulative amounts of cigarettes smoked by an individual and measures a smoker’s level of tobacco dependence, with a higher SI indicating higher levels of tobacco dependence, and is therefore helpful in understanding the intensity of an individual’s smoking and its potential health impacts (8). In a prospective study of smoking and disease risk from China, smoking was found to be associated with the risk of nine categories of disease, including infectious and parasitic diseases, neoplasms, endocrine, nutritional and metabolic disorders, circulatory disorders, respiratory disorders, gastrointestinal disorders, diseases of the skin and subcutaneous tissues, other signs, symptoms, and abnormal findings, as well as injuries, poisonings, and other external causes (9). Smoking aggravates skin aging, psoriasis, eczema, hidradenitis suppurativa, acne, lupus erythematosus, hair loss, and many other skin diseases (10).

Currently, a large number of studies have shown that smoking aggravates the skin manifestations of psoriasis, but research on smoking and nail psoriasis remains limited. Most studies on nail psoriasis focus on the number of nails affected, ignoring the area of the nail affected. Furthermore, most studies on smoking and nail psoriasis usually focus on comparing two groups of people: those with nail psoriasis and those without nail psoriasis, ignoring studies on smoking status and smoking intensity in a single group of people with nail psoriasis. In clinical practice, we have also found that nail psoriasis seems to be more severe in patients who smoke, so we speculate that smoking aggravates the severity of nail psoriasis and that the higher the smoking intensity, the more severe the nail psoriasis. In addition, nail involvement was mentioned earlier as a predictor of PsA, but studies have shown that smoking is negatively correlated with PsA. The effects of nail involvement and smoking on PsA seem to be contradictory, which makes us curious about how smoking affects nail psoriasis.

Therefore, in this study, we conducted a cross-sectional survey covering more than 1,000 hospitals in China. The aim is to understand the smoking rate among patients with nail psoriasis and to explore the association between smoking status, smoking intensity, and the severity of nail psoriasis, and between these smoking measures and the prevalence of PsA among patients with nail psoriasis. The study of potential factors is crucial for the diagnosis and treatment of nail psoriasis and the prevention of the development of psoriatic arthritis, and it helps to change the bad habits of patients.

## Materials and methods

### Study population

The data for this study were obtained from the National Clinical Research Centre for Skin and Immunological Diseases (NCRCSID) in China. In 2020, the NCRCSID conducted a multicenter observational study of psoriasis patients in China, collecting real-world data on psoriasis patients from more than 1,000 hospitals across China. This study recruited psoriasis patients with informed consent. All patients met the clinical criteria for psoriasis. Standardized questionnaires were used to collect information from psoriasis patients. The main contents of the questionnaire are as follows: (1) demographic characteristics: name, gender, age, marital status, educational attainment, health insurance, body mass index (BMI), etc.; (2) smoking status: current/former smokers, number of years of smoking, number of cigarettes smoked per day, smoking cessation status; (3) information on the onset and diagnosis of psoriasis: number and condition of affected nails, PEST score, DLQI score, PASI score, treatment needs, type of psoriasis, seasonality of psoriasis exacerbation, and family history of psoriasis.

Ethical approval was obtained from the Ethics Committee of Ningbo First Hospital (2022–028RS-01). This study strictly adhered to the Declaration of Helsinki.

### Diagnosis, inclusion, and exclusion criteria for nail psoriasis

This study conducted clinical diagnoses of psoriasis, and all psoriasis patients met the Chinese clinical dermatology diagnostic criteria for psoriasis and the guidelines for the diagnosis and treatment of psoriasis (11). A total of 7,037 psoriasis patients were initially identified. The inclusion criteria for this study were patients with psoriasis who had nail involvement and were over 18 years of age. Exclusion criteria included patients with positive fungal microscopy of the nails, mental disorders, neurological diseases, inability to provide informed consent, missing information, and rheumatic diseases (such as rheumatoid arthritis, ankylosing spondylitis, etc.) that could affect nail lesions and joint lesions. Based on the above criteria, a total of 1,044 patients were included in this study. In addition, based on the above exclusion criteria, we investigated the smoking situation of 4,892 adult psoriasis patients without nail psoriasis.

Among the 1,044 patients with nail psoriasis in this study, 118 had PSA, of whom 102 had a prior confirmed diagnosis of PsA and 12 were newly diagnosed with PsA. For patients newly diagnosed with PsA, the primary basis for diagnosis was a comprehensive assessment of the following factors: personal history of psoriasis, family history of psoriasis, nail involvement, and joint involvement. A minority of patients had imaging findings to support the diagnosis.

### Definition and index calculation

In this study, participants were categorized into non-smokers, former smokers, and smokers based on their smoking status. Smokers were defined as those who had smoked more than 100 cigarettes in their lifetime, while former smokers were defined as those who had quit smoking for at least 1 month. The smoking data collected included the number of cigarettes smoked per day and the cumulative number of years smoked. The smoking index = number of cigarettes smoked per day × cumulative number of years smoked. Based on the smoking index, current smokers were classified into three categories: mild smokers (smoking index ≤ 200), moderate smokers (200 < smoking index < 400), and severe smokers (smoking index ≥ 400).

In this study, the height and weight of patients with nail psoriasis were recorded, and the body mass index (BMI) was calculated using the formula BMI = weight (kg)/height (m^2^). Based on BMI, patients with nail psoriasis were classified as underweight (< 18.5), normal weight (18.5–23.9), overweight (24.0–27.9), and obese (≥ 28.0).

In this study, the Psoriasis Area and Severity Index (PSAI) score was used to assess the severity of psoriasis lesions in patients. The area and severity of lesions were assessed in four regions: the head and neck, trunk, upper limbs, and lower limbs. The severity of lesions was comprehensively evaluated based on erythema (E), scaling (D), and infiltration (I) of the lesions to obtain scores. The PASI score for each region was obtained by multiplying the score by the lesion area. The final PASI total score calculation formula is: head and neck lesion area × (E + I + D) × 0.1 + upper limb lesion area × (E + I + D) × 0.2 + trunk lesion area × (E + I + D) × 0.3 + lower limb lesion area × (E + I + D) × 0.4. The higher the PSAI score, the more severe the skin lesion. The Body Surface Area (BSA) score uses the patient’s palm (which accounts for approximately 1% of the body’s surface area) as the measurement area to score the patient’s total body skin lesions. A higher score indicates a more severe condition. The Psoriasis Epidemiology Screening Tool (PEST) questionnaire is an assessment tool used for rapid screening of patients for psoriatic arthritis. It consists of five questions, with each question scoring 1 point for a “yes” response, for a total score of 5 points. The Dermatology Life Quality Index (DLQI) score is a measure of quality of life for psoriasis patients, primarily assessing the impact of psoriasis on daily activities, dressing, leisure activities, work and school, interpersonal relationships, and treatment. A higher score indicates a greater impact of psoriasis on daily life. To analyze the extent of nail psoriasis involvement, the area of involvement of each nail was divided into three levels: < 50% area (< 50% nail involvement count), 50–90% area (50–90% nail involvement count), and > 90% (complete) area (> 90% nail involvement count). The number of nails in each level was counted and then summed to give the total number of nails involved in each patient.

In this study, we classified the severity of psoriasis based on the scores of the PASI, BSA and DLQI according to the Chinese Guidelines for the Diagnosis and Treatment of Psoriasis (2023 Edition). PASI < 3 points, BSA < 3%, or DLQI < 6 points are considered mild, PASI 3–10 points, BSA 3–10%, or DLQI 6–10 points are considered moderate, and PASI ≥ 10 points, BSA ≥ 10%, or DLQI ≥ 10 points are considered severe (11). The PEST score is used to screen for psoriatic arthritis. A score of 3 or higher is considered to indicate a high risk of psoriatic arthritis. In contrast, a score of less than 3 is considered to indicate a low risk of psoriatic arthritis.

### Data analysis

Data analysis was performed using statistical software SPSS (version 29.0). Qualitative and quantitative analyses were conducted on the clinical data obtained. For non-normally distributed quantitative variables, median and interquartile range (IQR) were used for representation, and intergroup differences were assessed using non-parametric rank-sum tests, with *post hoc* pairwise comparisons conducted by the Mann-Whitney U test. Qualitative variables were expressed as frequencies and proportions (%). Differences between groups for categorical data were assessed using chi-square tests, with *post hoc* pairwise comparisons performed using the Bonferroni method when significant differences were found. To study the relationship between smoking and the severity of nail psoriasis (PASI, PEST, number of affected nails, etc.), Spearman’s correlation analysis was used to assess the correlation between the two, and logistic regression was used to calculate the odds ratio (OR) and 95% confidence interval (95% CI) to assess the influencing factors of psoriatic arthritis. *P* < 0.05 differences were considered statistically significant.

## Results

### Smoking situation and sociodemographic characteristics of patients with nail psoriasis

First, in Table 1, we found that the proportion of males with nail psoriasis was higher than that of males without nail psoriasis (*P* < 0.001). We studied the smoking status of patients with or without nail psoriasis and found that the proportion of non-smokers among patients with nail psoriasis was 58.0%, which was lower than that among patients without nail psoriasis (74.1%). The proportion of smokers among patients with nail psoriasis was 34.6%, which was much higher than that among patients without nail psoriasis (20.2%). All of the above are statistically significant (*P* < 0.001). In the study of smoking intensity, we found that the proportion of mild smokers, moderate smokers, and severe smokers among patients with nail psoriasis was much higher than that among patients without nail psoriasis, and differences were statistically significant (*P* = 0.001).

**TABLE 1:** Smoking situation with or without nail psoriasis.

Variables	Psoriasis patients without nail psoriasis (*n* = 4892)	Psoriasis patients with nail psoriasis (*n* = 1044)	*P*-value
Gender, [*n*(%)]			< 0.001
Male	3,038(62.1)	774(74.1)	
Female	1,854(37.9)	270(25.9)	
Smoking status, [*n*(%)]			< 0.001
Non-smoker	3,656(74.7)	606(58.0)	
Former smoker	2,50(5.1)	77(7.4)	
Current smoker	9,86(20.2)	361(34.6)	
Smoking intensity, [*n*(%)]			0.001
Mild smoker	569(11.6)	182(50.4)	
Moderate smoker	134(2.7)	65(18.0)	
Severe smoker	925(18.9)	114(31.6)	

In this study, we included a total of 1,044 patients with nail psoriasis. The median age of these 1,044 patients was 42 years (IQR: 32.0–53.8), with 74.1% being male and 25.9% female. Among them, 80.7% were married, 60.9% were employed full-time, only 31.8% had received a bachelor’s degree or higher education, and only 21.9% had a family history of psoriasis. The median BMI was 24.3 kg/m^2^ (IQR: 22.3–26.7), with more than half of the patients having a BMI classified as overweight or obese. In terms of patients’ medical insurance coverage, the most common type is urban and rural employee medical insurance (41.9%), followed by urban and rural resident medical insurance (35.9%), with other types of medical insurance accounting for a very small proportion.

We divided 1,044 patients with nail psoriasis into three smoking status groups: non-smokers (*n* = 606, 58.0%), former smokers (*n* = 77, 7.4%), and smokers (*n* = 361, 34.6%). We further subdivided them into three smoking intensity groups based on their smoking index: mild smokers (*n* = 182, 50.4%), moderate smokers (*n* = 65, 18.0%), and severe smokers (*n* = 114, 31.6%). The data in Table 2 show that the median age of former smokers was higher than that of the non-smokers and current smokers (*P* < 0.001). The age differences among the three groups with varying smoking intensities were statistically significant (*P* < 0.001). The proportion of men among former smokers and smokers was higher than that among women (*P* < 0.001). The proportion of former smokers and smokers with a high school education or below was higher than that among non-smokers. Among non-smokers, 38% had a bachelor’s degree or above, and the difference was statistically significant (*P* < 0.001). In the three groups of smoking intensity, smoking intensity was found to be negatively correlated with educational attainment (*P* = 0.029). The higher the smoking intensity, the higher the proportion of people with a high school education or below, and the lower the proportion of people with a bachelor’s degree or above. Interestingly, we observed statistically significant differences in both smoking intensity and marital status among patients with nail psoriasis (*P* < 0.001). The higher the smoking intensity, the higher the proportion of married people. Additionally, a statistically significant difference was observed in smoking intensity across different occupation statuses (*P* < 0.05).

**TABLE 2:** Sociodemographic characteristics of patients with nail psoriasis.

Variables	Total nail psoriasis patients (*n* = 1044)	Patient with different tobacco smoking status				Patient with different tobacco smoking intensity			
		Non-smoker (*n* = 606)	Former smoker (*n* = 77)	Current smoker (*n* = 361)	*P*	Mild smoker (*n* = 182)	Moderate smoker (*n* = 65)	Severe smoker (*n* = 114)	*P*
Age, [median (IQR)]	42.0 (32.0–53.8)	40.0 (32.0–53.0)	52.0 (40.0–59.0)	43.0 (33.0–53.0)	< 0.001	35.0 (29.0–47.0)	44.0 (33.5–54.3)	51.0 (44.8–58.0)	< 0.001
Gender, [*n*(%)]					< 0.001				0.867
Male	774 (74.1)	353(58.3)	74(96.1)	347(96.1)		174(95.6)	63(96.9)	110(95.5)	
Female	270(25.9)	253(41.7)	3(3.9)	14(3.9)		8(4.4)	2(3.1)	4(3.5)	
Education, [*n*(%)]					< 0.001				0.029
Junior high and lower	320(30.7)	164(27.1)	33(42.9)	123(34.1)		55(30.2)	17(26.2)	51(44.7)	
Senior high	392(37.5)	212(35.0)	24(31.2)	156(43.2)		79(43.4)	31(47.7)	46(40.4)	
College and above	332(31.8)	230(38.0)	20(26.0)	82(24.7)		48(26.4)	17(26.2)	17(14.9)	
Family history, [*n*(%)]					0.070				0.679
Yes	229(21.9)	118(19.5)	18(23.4)	93(25.8)		49(26.9)	18(27.7)	26(22.8)	
No	815(78.1)	448(80.5)	59(76.6)	268(74.2)		133(73.1)	47(72.3)	88(77.2)	
BMI (kg/m^2^), [median (IQR)]	24.3 (22.3–26.7)	24.2 (22.2–26.6)	24.8 (22.4–27.2)	24.5 (22.3–26.9)	0.250	24.2 (22.3–26.7	24.3 (22.2–27.1)	25.0 (22.6–26.5)	0.854
BMI (kg/m^2^), [n(%)]					0.619				0,234
< 18.5 (lower body weight)	34(3.3)	22(3.6)	1(1.3)	11(3.0)		7(3.8)	1(1.5)	3(2.6)	
18.5–23.9 (normal body weight)	441(42.2)	256(42.2)	31(40.3)	154(42.7)		78(42.9)	31(47.7)	45(39.5)	
24.0–27.9 (overweight)	385(36.9)	231(38.1)	30(39.0)	124(34.3)		54(29.7)	21(32.3)	49(43.0)	
≥ 28 (obesity)	182(17.6)	97(16.0)	15(19.5)	72(19.9)		43(23.6)	12(18.5)	17(14.9)	
Marital status, [*n*(%)]					0.417				< 0.001
Unmarried	202(19.3)	125(20.6)	12(15.6)	65(18.0)		48(26.4)	9(13.8)	8(7.0)	
Married	842(80.7)	481(79.4)	65(84.4)	296(82.0)		134(73.6)	56(86.2)	106(93.0)	
Occupation status, [n(%)]					0.102				0.014
Full-time employment	636(60.9)	364(60.1)	47(61.0)	225(62.3)		119(65.4)	43(66.2)	63(55.3)	
Part-time employment	60(5.7)	36(5.9)	3(3.9)	21(5.8)		15(8.2)	2(3.1)	4(3.5)	
Unemployment	143(13.7)	79(13.0)	8(10.4)	56(15.5)		23(12.6)	9(13.8)	24(21.1)	
Student	31(3.0)	25(4.1)	0(0.0)	6(1.7)		6(3.3)	0(0.0)	0(0,0)	
Retired	174(16.7)	101(16.8)	19(24.7)	53(14.7)		19(10.4)	11(16.9)	23(20.2)	
Medical insurance type, [n(%)]					0.685				0.240
Urban employee basic MI	572(49.1)	339(49.3)	45(52.3)	188(47.8)		87(44.8)	43(55.8)	58(47.5)	
Urban-rural resident basic MI	402(34.5)	227(33.0)	27(31.4)	148(37.7)		82(42.3)	19(24.7)	47(38.5)	
Supplementary MI	13(1.1)	8(1.2)	0(0.0)	5(1.3)		0(0.0)	2(2.6)	3(2.5)	
Government-funded MI	37(3.2)	24(3.5)	2(2.3)	11(2.8)		6(3.1)	2(2.6)	3(2.5)	
Non-local MI	32(2.7)	19(2.8)	3(3.5)	10(2.5)		5(2.6)	2(2.6)	3(2.5)	
Commercial HI	47(4.0)	32(4.7)	3(3.5)	12(3.1)		5(2.6)	4(5.2)	3(2.5)	
MI for psoriasis	17(1.5)	12(1.7)	3(3.5)	2(0.5)		2(1.0)	0(0.0)	0(0,0)	
Other MI types	45(3.9)	26(3.8)	3(3.5)	17(4.3)		7(3.6)	5(6.5)	5(4.1)	

Supplementary medical insurance: medical insurance other than commercial insurance (such as supplementary medical insurance provided by enterprises and social mutual aid).

### The relationship between smoking and the severity of nail psoriasis

**FIGURE 1:**
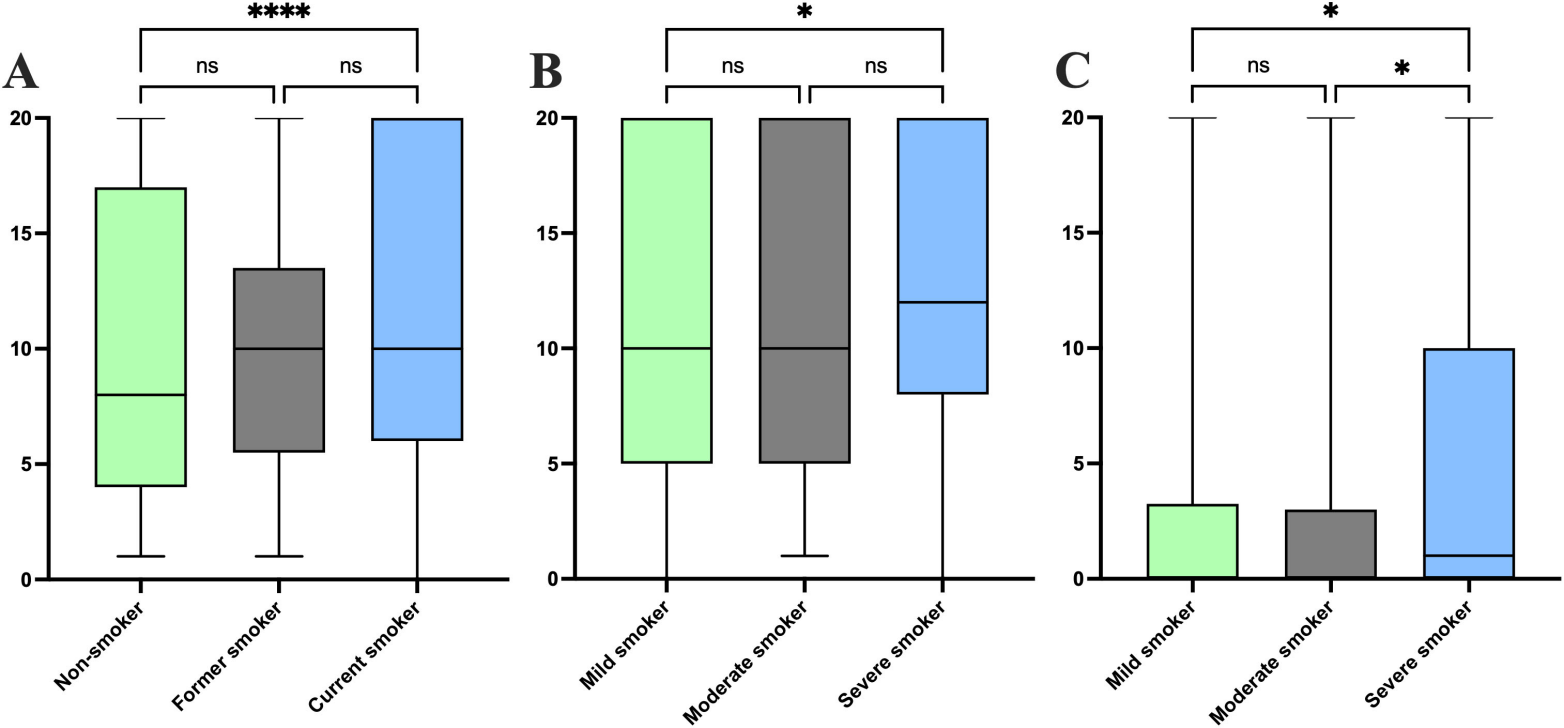
**(A)** Relationship between tobacco smoking status and total nail involvement count. **(B)** Relationship between tobacco smoking intensity and total nail involvement count. **(C)** Relationship between tobacco smoking intensity > 90% nail involvement count. *P < 0.05, ****P < 0.0001, ns, no statistical significance.

This study examined the effects of smoking status and smoking intensity separately on the severity of nail psoriasis. In exploring the impact of smoking status on the severity of nail psoriasis, we divided smoking status into three groups: non-smokers (*n* = 606), former smokers (*n* = 82), and smokers (*n* = 384). Our analysis revealed no statistically significant differences between smoking status and disease severity metrics in patients with nail psoriasis (*P* > 0.05), including PASI score (*P* = 0.316), BSA score (*P* = 0.781), DLQI score (*P* = 0.108), and PEST score (*P* = 0.758), as shown in Table 3. However, smoking status showed a significant difference with total nail involvement count (*P* < 0.001). Our analysis demonstrated that current smokers had significantly higher nail involvement counts than non-smokers (*P* < 0.0001), while no significant difference was found between former smokers and non-smokers (*P* = 0.364), nor between former smokers and current smokers (*P* = 0.100) (Figure 1A). No significant differences were observed in the < 50% nail involvement count (*P* = 0.791), 50–90% nail involvement count (*P* = 0.650), or > 90% nail involvement count (*P* = 0.439) across smoking status groups (Table 3), although the median total nail involvement count was numerically higher in current smokers than in non-smokers.

**TABLE 3:** Relationship between smoking status and severity of nail psoriasis.

Variables	Non-smoker (*n* = 606)	Former smoker (*n* = 77)	Current smoker (*n* = 361)	*P*-value
PASI score, [median (IQR)]	11.8(5.8–19.8)	13.9(5.9–27.2)	12.7(6.3–22.4)	0.316
BSA score, [median (IQR)]	19.0(6.0–42.0)	15.0(5.0–47.5)	20.0(7.8–41.5)	0.781
DLQI score, [median (IQR)]	10.0(5.0–16.3)	7.0(2.0–16.0)	10.0(4.0–15.0)	0.108
PEST score, [median (IQR)]	1.0(0.0–2.0)	1.0(0.5–2.0)	1.0(0.0–2.0)	0.758
PEST risk level, [*n*(%)]				0.264
Low-risk PEST	488(80.5)	67(87.0)	301(83.4)	
High-risk PEST	118(19.5)	10(13)	60(16.6)	
Total nail involvement count, [median (IQR)]	8.0(4.0–17.0)	10.0(5.5–13.5)	10.0(6.0–20.0)	< 0.001
<50% nail involvement count, [median (IQR)]	3.0(1.0–6.0)	2.0(0.0–6.5)	3.0(0.0–8.0)	0.791
50–90% nail involvement count, [median (IQR)]	0.0(0.0–4.0)	0.0(0.0–4.0)	0.0(0.0–5.0)	0.650
> 90% nail involvement count, [median (IQR)]	0.0(0.0–3.0)	0.0(0.0–4.0)	0.0(0.0–5.0)	0.439

In exploring the impact of smoking intensity on the severity of nail psoriasis, we divided smoking intensity into three groups: mild smokers (*n* = 182), moderate smokers (*n* = 65), and severe smokers (*n* = 114). Analysis revealed no significant differences between smoking intensity and disease severity metrics (*P* > 0.05), including the PASI score (*P* = 0.390), BSA score (*P* = 0.255), and DLQI score (*P* = 0.091). Smoking intensity showed statistically significant differences in both PEST scores (*P* = 0.022) and PEST risk levels (*P* = 0.007), as shown in Table 4. Moderate smokers had higher PEST score than both mild and severe smokers, along with a greater proportion of high PEST risk. In addition, there were significant differences between smoking intensity and total nail involvement count (*P* = 0.015), with a significant difference specifically between mild and severe smokers (*P* = 0.004) (Figure 1B). No significant differences were observed between smoking intensity and both < 50% nail involvement count (*P* = 0.250) and 50–90% nail involvement count (*P* = 0.561). However, we found that the median and quartile of > 90% nail involvement count in severe smokers were higher than those in mild smokers (*P* = 0.005) and moderate smokers (*P* = 0.009) (Figure 1C).

**TABLE 4:** Relationship between smoking intensity and severity of nail psoriasis.

Variables	Mild Smoker (*n* = 182)	Moderate smoker (*n* = 65)	Severe smoker (*n* = 114)	*P*-value
PASI score, [median (IQR)]	12.8(6.5–22.8)	11.9(5.4–18.5)	14.0(6.5–22.9)	0.390
BSA score, [median (IQR)]	20.0(7.0–43.3)	16.0(5.5–35.0)	20.0(8.0–50.0)	0.255
DLQI score, [median (IQR)]	11.5(4.0–17.3)	10.0(4.0–13.0)	9.0(4.0–14.3)	0.091
PEST score, [median (IQR)]	1.0(0.0–2.0)	1.0(1.0–3.0)	1.0(0.0–2.0)	0.022
PEST risk level, [*n*(%)]				0.007
Low-risk PEST	154(84,6)	46(70.8)	101(88.6)	
High-risk PEST	28(15.4)	19(29.2)	13(11.4)	
Total nail involvement count, [median (IQR)]	10.0(5.0–20.0)	10.0(5.0–20.0)	12.0(8.0–20.0)	0.015
< 50% nail involvement count, [median (IQR)]	3.0(0.0–8.0)	4.0(1.0–9.0)	2.0(0.0–7.0)	0.250
50–90% nail involvement count, [median (IQR)]	0.0(0.0–0.0)	1.0(0.0–6.0)	0.0(0.0–4.3)	0.561
> 90% nail involvement count, [median (IQR)]	0.0(0.0–3.3)	0.0(0.0–3.0)	1.0(0.0–10.0)	0.005

*P* < 0.05 is statistically significant. If not annotated, it means *P* > 0.05.

**FIGURE 2:**
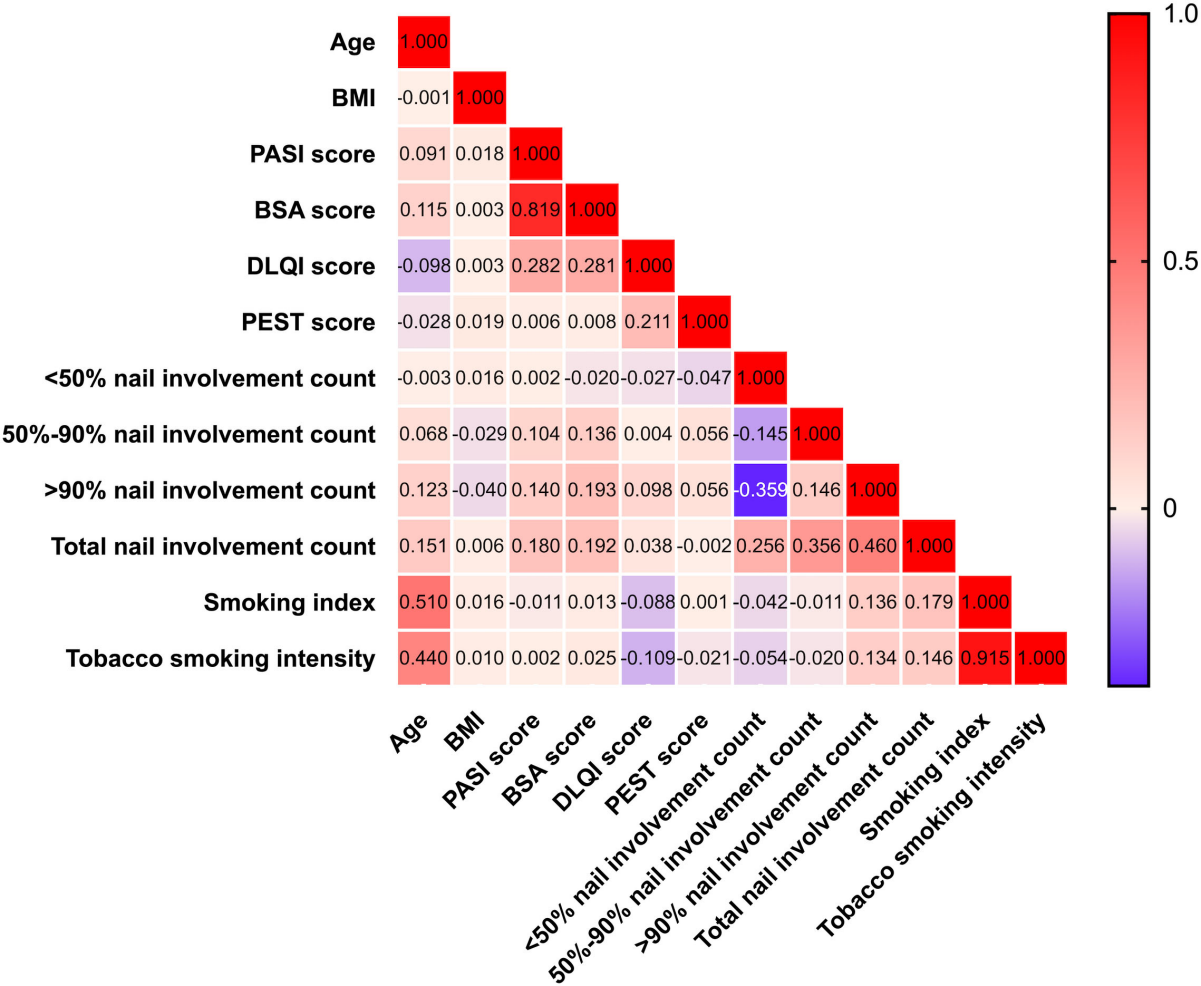
Heat map showing the correlation coefficient between smoking intensity and nail psoriasis severity.

As shown in Figure 2, Spearman correlation analysis revealed weak positive correlations between smoking intensity and total nail involvement count (*r* = 0.146) as well as > 90% nails involvement count (*r* = 0.134). Similarly, the smoking index showed weak positive correlations with total nail involvement count (*r* = 0.179) and > 90% nails involvement count (*r* = 0.136). In contrast, neither smoking intensity nor smoking index correlated with PEST, PASI, or BSA scores. In addition, we observed a weak negative correlation between smoking intensity and DLQI score (*r* = −0.139) in patients with nail psoriasis, indicating that higher smoking intensity was associated with lower DLQI scores. While previous studies reported positive correlations between smoking intensity and DLQI score in patients with cutaneous psoriasis only (12, 13), our findings in nail psoriasis patients demonstrate an opposite trend. Additionally, age showed weak positive correlations with PASI score (*r* = 0.091), BSA score (*r* = 0.115), 50–90% nail involvement count (*r* = 0.068), > 90% nail involvement count (*r* = 0.123), and total nail involvement count (*r* = 0.151). Moderate positive correlations were found between age and smoking intensity (*r* = 0.440) as well as smoking index (*r* = 0.510), while a weak negative correlation was observed with DLQI score (*r* = −0.098). Both PASI and BSA scores demonstrated weak positive correlations (0.00 < *r* < 0.20) with 50–90% nail involvement count, > 90% nail involvement count, and total nail involvement count. DLQI score showed only a weak positive correlation with > 90% nail involvement count (*r* = 0.098), while PEST score showed no correlation with any nail involvement parameters.

### The relationship between smoking and nail psoriasis, accompanied by psoriatic arthritis

As PEST risk level functions as an early screening tool for identifying PsA risk in psoriasis patients, we examined the potential impact of smoking on this screened cohort. As shown in Table 5, a statistically significant difference was observed between PEST risk level and smoking intensity (*P* = 0.007). *Post hoc* comparisons revealed that the proportion of moderate smokers was significantly higher in the high-PEST-risk group than in the low-risk group (*P* < 0.05), although no significant difference was observed in the distribution of mild and severe smokers across PEST risk levels (*P* > 0.05). And, no significant difference was observed between PEST risk levels and smoking status (*P* = 0.264). We found that nail psoriasis patients with PsA showed statistically significant differences in age (*P* = 0.004), DLQI score (*P* = 0.002), DLQI severity (*P* = 0.002), BSA score (*P* = 0.042), > 90% nails involvement count (*P* < 0.001), PEST score (*P* < 0.001), and PEST risk level (*P* < 0.001) compared to those without PsA. Analysis revealed a statistically significant difference between the presence of PsA and smoking status. *Post hoc* comparisons revealed that the proportion of non-smokers was significantly higher in patients with PsA than in those without PsA (*P* < 0.05), while the proportion of smokers was significantly lower in the PsA group (*P* < 0.05). However, the difference between the presence of PsA and smoking intensity was not statistically significant (*P* = 0.135).

**TABLE 5:** PEST risk level or factors affecting psoriatic arthritis.

Variables	PEST risk level			Presence of PsA		
	Low-risk PEST (*n* = 856)	High-risk PEST (*n* = 188)	*P*-value	No (*n* = 926)	Yes (*n* = 118)	*P*-value
Age,[median (IQR)]	42.0(32.0–53.8)	44.0(34.0–53.75)	0.129	42.0(32.0–53.0)	46.5(36.0–57.3)	0.004
Gender, [*n*(%)]			0.123			0.058
Female	213(24.9)	57(30.3)		231(24.9)	39(33.1)	
Male	643(75.1)	188(69.7)		695(75.1)	79(66.9)	
Family history, [*n*(%)]			0.963			0.165
No	668(78.0)	147(78.2)		717(77.4)	98(83.1)	
Yes	188(22.0)	41(21.8)		209(22.6)	20(16.9)	
BMI, [median (IQR)]	24.2(22.2–26.7)	24.3(22.5–26.9)	0.760	24.4(22.3–26.8)	24.1(22.1–25.7)	0.191
BMI, [*n*(%)]			0.253			0.390
< 18.5 (lower body weight)	24(2.8)	10(5.3)		29(3.1)	5(4.2)	
18.5–23.9 (normal body weight)	369(43.1)	72(38.8)		392(42.3)	49(41.5)	
24.0–27.9 (overweight)	312(36.4)	73(38.8)		336(36.3)	49(41.5)	
≥ 28 (obesity)	151(17.6)	33(17.6)	169(18.3)	15(12.7)
DLQI score, [median (IQR)]	9.0(4.0–15.0)	14.0(7.0–21.0)	< 0.001	9.0(4.0–16.0)	12.0(6.0–20.0)	0.002
DLQI severity, [*n*(%)]			< 0.001			0.022
Mild DLQI	278(32.5)	37(19.7)		288(31.1)	27(22.9)	
Moderate DLQI	178(20.8)	21(11.2)		182(19.7)	17(14.4)	
Severe DLQI	400(46.7)	130(69.1)		456(49.2)	74(62.7)	
PASI score, [median (IQR)]	12.0(5.8–21.6)	14.2(6.6–20.0)	0.482	12.3(6.0–21.6)	12.5(5.7–18.7)	0.652
PASI severity, [n(%)]			0.756			0.548
Mild PSAI	105(12.3)	24(12.8)		113(12.2)	16(13.6)	
Moderate PASI	251(29.3)	50(26.6)		272(29.2)	24(24.6)	
Severe PASI	500(58.4)	114(60.6)		541(58.4)	73(61.9)	
BSA score, [median (IQR)]	18.9(6.8–40.0)	20.0(8.0–52.0)	0.121	18.5(6.4–40.0)	24.5(9.8–50.5)	0.042
BSA severity, [n(%)]			0.211			0.053
Mild BSA	72(8.4)	21(11.2)		80(8.6)	13(11.0)	
Moderate BSA	197(23.0)	34(18.1)		215(23.2)	16(13.6)	
Severe BSA	587(68.6)	133(70.7)		631(68.1)	89(75.4)	
Total nail involvement count, [median (IQR)]	10.0(4.0–20.0)	10.0(5.0–19.0)	0.935	10.0(4.0–20.0)	9.0(5.0–14.5)	0.742
< 50% nail involvement count, [median (IQR)]	3.0(0.0–6.75)	2.0(0.0–6.0)	0.206	3.0(0.0–7.0)	2.0(0.75–5.0)	0.121
50–90% nail involvement count, [median (IQR)]	0.0(0.0–4.0)	1.0(0.0–4.8)	0.307	0.0(0.0–4.0)	2.0(0.0–4.0)	0.014
> 90% nail involvement count, [median (IQR)]	0.0(0.0–3.0)	1.0(0.0–3.8)	0.043	0.0(0.0–3.0)	2.0(0.0–4.0)	< 0.001
Tobacco smoking status, [*n*(%)]			0.264			0.028
Non-smoker	488(57.0)	118(62.8)		524(56.6)	82(69.5)	
Former-smoker	67(7.8)	10(5.3)		71(7.7)	6(5.1)	
Current-smoker	301(35.2)	60(31.9)		331(35.7)	30(25.4)	
Smoking index, [median (IQR)]	200.0(100.0–400.0)	222.5(125.0–357.5)	0.808	200.0(100.0–400.0)	210.0(100.0–305.0)	0.551
Tobacco smoking intensity, [*n*(%)]			0.007			0.135
Mild smoker	154(51.2)	28(46.7)		167(50.5)	15(50.0)	
Moderate smoker	46(15.3)	19(31.7)		56(16.9)	9(30.0)	
Severe smoker	101(33.6)	13(21.7)		108(32.6)	6(20.0)	
PEST score, [median (IQR)]				1.0(0.0–2.0)	3.5(1.8–4.3)	< 0.001
PEST risk level, [*n*(%)]						< 0.001
Low-risk PEST				291(87.9)	10(33.3)
High-risk PEST				40(12.1)	20(66.7)

*P* < 0.05, statistically significant, *P* < 0.01, obviously statistically significant. If not annotated, it means *P* > 0.05.

**FIGURE 3:**
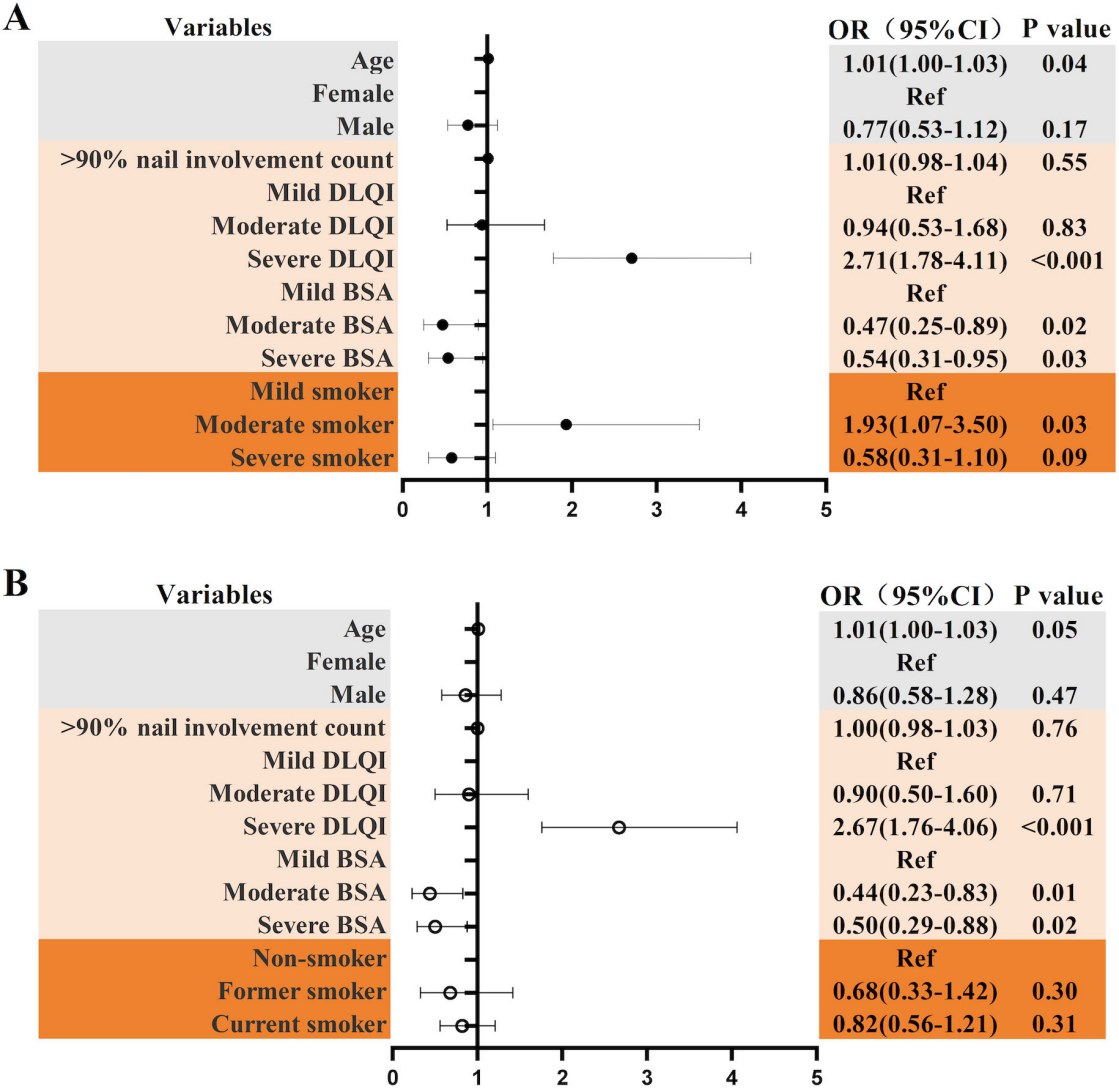
**(A)** Logistic analysis of smoking intensity and PEST risk level. **(B)** Logistic analysis of smoking status and PEST risk level; *P* < 0.05 is statistically significant.

Based on Table 5, we selected partial influencing factors for both PEST risk level and the presence of PSA for logistic regression analysis. In the correlation analysis of PEST risk level, we selected independent variables with *P*-values less than 0.2 from Table 5 for binary logistic regression analysis. In Figure 3A, we found > 90% nail involvement count was not associated with PEST risk level. The OR for age was 1.01 (95% CI: 1.00–1.03). Compared with the mild DLQI, the severe DLQI had an OR of 2.71 (95% CI: 1.78–4.11). Compared with mild BSA, moderate and severe BSA showed a negative correlation. The OR for moderate BSA was 0.47 (95% CI: 0.25–0.89), and the OR for severe BSA was 0.54 (95% CI: 0.31–0.95). Compared with mild smokers, moderate smokers had an OR of 1.93 (95% CI: 1.07–3.50), but there was no significant difference between severe smokers and mild smokers (*P* > 0.05).

Although smoking status was not significantly associated with PEST risk levels (*P* = 0.264), we proceeded with logistic regression to further explore this relationship as part of our analytical strategy. In this model, the OR for age was 1.01 (95% CI: 1.00–1.03), the OR for severe DLQI was 2.67 (95% CI: 1.76–4.06), the OR for moderate BSA was 0.44 (95% CI: 0.23–0.83), and the OR for severe BSA was 0.50 (95% CI: 0.29–0.88). No association was found between smoking status and PEST risk level, as shown in Figure 3B.

**FIGURE 4:**
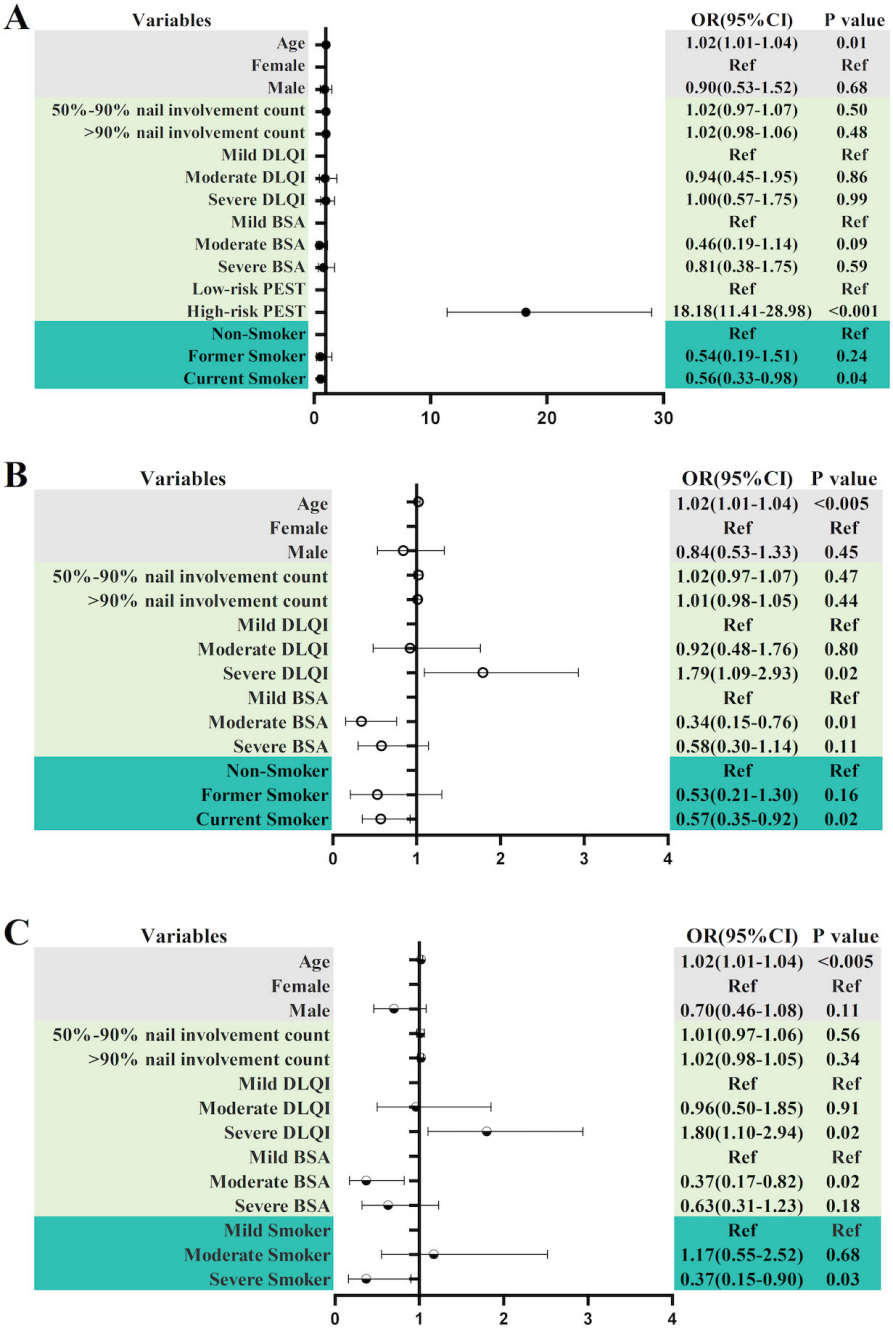
**(A)** Logistic regression analysis of smoking status before adjustment and PsA status. **(B)** Logistic regression analysis of smoking status after adjustment and PsA status. **(C)** Logistic regression analysis of smoking intensity and PsA status; *P* < 0.05 is statistically significant.

In the correlation analysis of the presence PsA, we selected the independent variables with a *P*-value less than 0.1 in Table 5 for binary logistic regression analysis. Although the *P*-values for PEST score and PEST risk level were both less than 0.01, there was a strong collinearity between PEST score and < 50% nail involvement count, 50–90% nail involvement count, > 90% nail involvement count and total nail involvement count, we decided not to include the PEST score in the logistic regression model. In Figure 4A, we found that the OR for age was 1.02 (95% CI: 1.01–1.04), and the high-risk level of PEST was a strong associated factor for whether or not a person had PsA, with an OR value of 18.18 (95% CI: 11.41–28.98), which was statistically significant (*P* < 0.001). Compared with non-smoking, smoking was a negative associated factor, with an OR of 0.56 (95% CI: 0.33–0.98). Former smoking had no statistical significance (*P* = 0.24).

The PEST score serves as a rapid screening tool for PsA and is recognized as a known independent influencing factor for PsA, thereby affecting other dependent variables (14). We further optimized the model by removing the PEST risk level as an influencing factor and then performing binary logistic regression. After removing the PEST risk level factor, we found that the OR for age was 1.02 (95% CI: 1.01–1.04), and smoking remained a negative association, with an OR of 0.57 (95% CI: 0.35–0.92). Compared with the mild DLQI, the severe DLQI was an exposure factor, with an OR of 1.79 (95% CI: 1.09–2.93). Compared with mild BSA, moderate BSA had an OR of 0.34 (95% CI: 0.15–0.76), which was a negative associated factor. However, there was no correlation between severe BSA and mild BSA (*P* > 0.05) (Figure 4B). Through the above research, we found that regardless of whether the strong influencing factor of PEST risk level was removed, age and smoking both affected the occurrence of psoriatic arthritis. In particular, smoking was a negative associated factor compared to not smoking, with an OR of less than 1, regardless of whether the strong associated factor was removed.

Based on previous evidence demonstrating a positive correlation between smoking intensity—particularly heavy smoking—and increased risk of PsA (15), we included smoking intensity in the multivariate logistic regression model for PsA risk assessment. This variable was retained due to its established clinical relevance, despite not achieving statistical significance (*P* = 0.14) in univariate analysis (Table 5). Therefore, we adjusted the logistic regression model again. In Figure 4C, we also found that whether or not a patient had PsA was still associated with age, severe DLQI, and moderate BSA, with OR (95% CI) of 1.02 (1.01–1.04), 1.10 (1.80–2.94), and 0.37 (0.17–0.82), respectively. Severe smokers were statistically significant compared with mild smokers (*P* = 0.03), with an OR of 0.37 (0.16–0.90), while moderate smokers were not statistically significant compared with mild smokers (*P* = 0.68). In this model, moderate BSA and severe smoking were still negative associated factors.

In summary, the logistic regression analysis revealed no association between smoking status and PEST risk level, while moderate smoking demonstrated a positive association with PEST risk level. However, both current smoking and heavy smoking showed negative associations with PsA development.

## Discussion

Nail involvement is a common site of involvement in psoriasis, and nail psoriasis is difficult to treat. Smoking is currently considered to be one of the important factors affecting psoriasis of the skin, but there has been insufficient research on nail psoriasis. Therefore, we explored the relationship between smoking and the demographics and severity of nail psoriasis.

Given the difference in smoking prevalence between men and women, we first conducted a gender-stratified analysis to assess potential confounding or effect modification. However, the small number of female smokers precluded a meaningful analysis in this subgroup. Since the results for smoking-related variables in men were similar to those in the full sample, we present the findings from the overall sample of 1,044 nail psoriasis patients. We found that the smoking rate among patients with nail psoriasis was 42.0%, with 34.6% currently smokers and 7.4% former smokers. Previous studies have also found that the smoking rate among patients with nail psoriasis is higher than that among patients with only plaque psoriasis. The proportion of smokers among nail psoriasis patients was higher than that among non-nail psoriasis patients, and the proportion of nail psoriasis patients with different smoking intensities was higher than that among non-nail psoriasis patients. For example, in a study of nail psoriasis in Egypt, the smoking rate was 44.2%, while the smoking rate among patients without nail psoriasis was 27.1% (16). We also found that the number of men with nail psoriasis far exceeded that of women, which is consistent with previous studies on nail psoriasis in Switzerland and Germany (17, 18). This may be related to the fact that men engage in more physical labor, which can lead to nail inflammation (16). In this study, the median PASI score for patients with nail psoriasis was 12.3 (IQR: 6.0–21.3), and the mean score was 15.8 (SD: 13.6), which is consistent with the findings of Bedair et al. and Tan et al. that the PASI score for patients with nail psoriasis was higher than the PSAI score for patients without nail psoriasis (16, 19). In this study, no statistically significant association was found between smoking status or smoking intensity and the skin psoriasis severity scores (PASI and BSA) or the DLQI score among patients with nail psoriasis (*P* > 0.05). Furthermore, Spearman correlation analysis revealed no significant correlation between either PASI score or BSA score and smoking intensity or smoking index. However, the DLQI score demonstrated a negative correlation with smoking intensity. This finding appeared inconsistent with previous studies, which, to our knowledge, have reported that smoking increases PASI score, BSA score, and DLQI score in patients with plaque psoriasis (20). In this study, smoking was found to not affect skin lesion scores in patients with nail psoriasis, and smoking intensity was even negatively correlated with DLQI score, which may be unprecedented. We speculate that smoking may relieve anxiety and stress in patients with nail psoriasis. In a study on stress coping methods in psoriasis patients, 41% of psoriasis patients who smoked said that smoking relieved their stress (21). It is also possible that smoking intensity is related to the number of years smoked. Patients who have smoked for a longer period tend to be older, and older patients with nail psoriasis have lower expectations for their appearance and social life than younger patients with nail psoriasis, resulting in a lower DLQI score (22). Of course, it is also possible that this is related to statistical anomalies, as there was no correlation between the smoking index and DLQI scores in this study (*P* > 0.05). Still, the smoking intensity derived from the smoking index was negatively correlated with DLQI scores, or it may be related to uncontrolled confounding factors. Therefore, further research is needed. In addition, Yu-Ting Peng et al. found that patients with nail psoriasis had higher PASI score, BSA score, and DLQI score than patients without nail psoriasis (23). Although there was no statistical significance between the severity of skin lesions and smoking in patients with nail psoriasis in this study, there was a statistical difference between the total nail involvement count and smoking status or smoking intensity in patients with nail psoriasis. The median total nail involvement count in smokers was higher than that in non-smokers, and *P* < 0.05. There was no statistically significant difference in the total nail involvement count between former smokers and non-smokers or smokers. The median total nail involvement count of severe smokers was also significantly higher than that of mild smokers and moderate smokers, and was statistically significant. Previous studies have also found that smoking significantly increases the incidence of nail psoriasis in patients with psoriasis (20, 24). Smoking may increase the severity of nail psoriasis because it induces oxidative stress and activates inflammatory pathways, thereby exacerbating nail bed inflammation, or because nicotine reduces blood flow to the nail bed, thereby reducing local drug penetration and therapeutic efficacy. It may also be caused by the Koebner phenomenon (20, 25). Age is negatively correlated with DLQI scores. We speculate that younger people may be more prone to depression due to psoriasis because psoriasis has a greater impact on the social and aesthetic needs of younger people than older people (22). Older people may gradually become accustomed to psoriasis due to its long-term effects.

Nail psoriasis is an independent risk factor for psoriatic arthritis, and smoking is a risk factor for psoriasis, but in some studies, smoking is negatively correlated with psoriatic arthritis (26). Therefore, to understand the effect of smoking on psoriatic arthritis in patients with nail psoriasis, we explored the effect of smoking on psoriatic arthritis in patients with nail psoriasis.

Among the 1,044 patients with nail psoriasis in this study, 188 (18.0%) were at high-risk PEST, and 118 (11.3%) had psoriatic arthritis. The prevalence of PsA was slightly lower than in previous studies. A meta-analysis study found that the prevalence of PsA in Asians was 14.0% (95% CI, 11.7–16.3%) (27). As shown in Figure 3, the OR for moderate smokers was 1.93 (95% CI: 1.07–3.50). Compared with mild smokers, moderate smokers were at a higher risk for the PEST risk level, but there was no correlation with severe smokers. We speculate that this may be because severe smokers have more severe skin lesions and seek treatment earlier and more aggressively (4), thereby reducing their PEST score, but this still needs further study. According to Figure 3, we found that among the factors affecting the PEST risk level, there was no correlation between former smokers or smokers and non-smokers. Mease et al. also found that PEST risk level was unrelated to smoking status (28). In addition, in the study of PEST risk level, compared with patients with mild DLQI, those with severe DLQI had an OR greater than 2, which was a strong risk signal for PEST risk level. This result is similar to that of a cross-sectional study from China (29). In this study, the BSA severity was negatively correlated with the PEST risk level, which contradicts the findings from studies on psoriasis of the skin, where BSA severity was positively correlated with the PEST risk level. The higher the BSA, the more likely a person is to develop PsA (30). This negative correlation in patients with nail psoriasis does not seem to have been clarified in previous studies, which may be related to selection bias or other confounding factors in this study. It is also possible that in the current era of biological agents, patients with a higher BSA score are more actively receiving systemic treatment. Further research is needed to clarify the reasons for this.

In our regression study on whether or not to suffer from PsA, we found that regardless of whether the strong correlation factor of PEST risk level was excluded, smokers had a lower risk of PsA than non-smokers, and smoking was a negative associated factor. Severe smokers had a lower risk of PsA than mild smokers. Severe smokers also became a negative associated factor. Previous studies have also found a negative correlation between smoking and the risk of psoriatic arthritis in patients with psoriasis (5, 31, 32). This phenomenon of smoking and psoriatic arthritis is called the “smoking paradox” (33). Although previous studies have mainly focused on the risk of psoriatic arthritis in patients with skin psoriasis who smoke, the patients with nail psoriasis in this study also fit this paradox. The emergence of this paradox may be due to the relatively low blood supply to the attachment points of psoriasis patients. Approximately 10% of patients experience changes in blood flow around the attachment points. The vasoconstrictive and anti-angiogenic effects of cigarette smoke may alleviate symptoms associated with PsA flare-ups, leading to the phenomenon of a negative correlation between smoking and PsA progression (34). Alternatively, this phenomenon may be caused by reduced expression of IL-1β and IL-8 in smokers and the toll-like receptor pathway’s response to infectious agents (26). Another theory suggests that nicotine in tobacco activates the α7 nicotinic acetylcholine receptor (α7nAChR), thereby inhibiting downstream pro-inflammatory signals and reducing joint inflammation (31). At the genetic level, the smoking paradox may be associated with polymorphisms in the IL-13 gene or with HLA-C*06 negativity (31, 35). Additionally, the observed negative correlation might be explained by the possibility that smoking’s effect on PsA risk is almost entirely mediated through its effect on psoriasis. In other words, when the study population is restricted to psoriasis patients, the observed association between smoking and PsA risk primarily reflects a direct effect. Consequently, traditional studies estimating the impact of smoking within psoriasis populations may capture only this direct effect, rather than the total effect of smoking on PsA risk (33). Or, due to the amplification effect of index event bias, limiting the study to psoriasis patients, the strong association between smoking and psoriasis amplifies unmeasured confounding factors (such as genetic, environmental, and socioeconomic factors), even if the confounding factors have a weak association with smoking and PsA, it is sufficient to reverse the observed effect direction (33). It is also possible that PsA patients may voluntarily quit smoking due to their symptoms and condition, thereby artificially lowering the smoking rate and intensity among PsA patients. Although there are currently no surveys or studies on smoking cessation intentions among psoriatic arthritis patients, a smoking cessation study among rheumatoid arthritis patients found that 43.4% of smoking patients with rheumatoid arthritis had intentions to quit smoking due to disease progression (36). In a study on lifestyle changes in psoriasis patients, it was found that among those willing to change their lifestyle, only 8.1% prioritized quitting smoking as their primary change (37). However, other studies have found that smoking exacerbates the development of PsA (38), so there is still controversy over whether smoking exacerbates the occurrence of PsA (39), and more in-depth research is needed. Severe DLQI is an exposure factor for whether or not a patient has PSA. A clinical study of secukinumab found that the combination of PsA was associated with a greater decline in quality of life, with a greater impact on itching, irritation, pain, and joint symptoms (40).

In summary, smoking affects nail psoriasis and psoriatic arthritis. Although this study suggests that smoking is negatively correlated with psoriatic arthritis, this phenomenon remains controversial. Therefore, we still recommend that patients with nail psoriasis and psoriatic arthritis control their smoking. Quitting smoking can reduce the risk of psoriasis (41) and is more beneficial for the treatment of psoriasis (42). In clinical practice, encouraging a healthy lifestyle remains an effective method in the treatment of the disease.

## Conclusion

Patients with nail psoriasis have higher smoking rates and smoking intensity compared to those without nail psoriasis. The total nail involvement count was higher in current smokers than in non-smokers. Smoking intensity was positively associated with total nail involvement count and individual nails with > 90% area involvement count. Current smoking was a negative associated factor for PEST risk level. Both current smoking and severe smoking were negative associations with the presence of psoriatic arthritis.

### Strengths and limitations

This study was a large-scale, multi-center clinical study in which all patients were assessed by professional dermatologists, making the results relatively more reliable. Unlike previous studies that assessed smoking based on smoking frequency, years of smoking, and number of cigarettes smoked, this study used a smoking index to quantify smoking and classified smoking intensity according to the smoking index, enabling a more accurate assessment of the impact of tobacco on nail psoriasis. This study used the nail involvement area to assess the severity of psoriatic nails, rather than simply counting the total nail involvement count. Although this study only observed the total nail involvement count and the area of nail involvement in nail psoriasis and did not use professional scoring to evaluate patients with nail psoriasis, in clinical practice, outpatient doctors often do not use cumbersome professional scoring to assess the condition of nails. On the contrary, the total nail involvement count and the area of nail involvement provide outpatient doctors with an intuitive impression, facilitating their quick understanding of the condition of patients with nail psoriasis. Therefore, the total nail involvement count and area statistics in this study are more in line with clinical practice.

This study has some limitations. The patients’ smoking history and weekly cigarette consumption were recorded based on their self-reported memories, which may be subject to recall bias. This study lacked information on secondhand smoke exposure, which may have underestimated the impact of tobacco on nail psoriasis to a certain extent. This study found that the diagnosis of PsA in newly diagnosed patients primarily relied on clinical manifestations and medical history, lacking support from laboratory tests and imaging evidence. This study only counted the number of nails with different areas of involvement and the total number of nails, and did not use more specialized nail psoriasis severity score (NAPSI) and Nijmegen nail psoriasis activity score (N-NAIL) to evaluate the patients in this study. This study did not perform statistical analysis on psoriasis patients without nail psoriasis, so there is a certain degree of limitation in that there are no statistical analysis results between nail psoriasis and psoriasis without nail psoriasis. In subsequent further studies, it is recommended to use a dermatoscope to assess lesions in the nail matrix and nail bed separately, and to use the results of the dermatoscope to thoroughly evaluate the impact of smoking on NAPSI and Nijimegen scores.

## Data Availability

The original contributions presented in the study are included in the article/supplementary material, further inquiries can be directed to the corresponding author.
